# Diagnostic and prognostic role of HE4 expression in multiple carcinomas: A protocol for systematic review and meta-analysis

**DOI:** 10.1097/MD.0000000000015336

**Published:** 2019-07-12

**Authors:** Chellan Kumarasamy, Madurantakam Royam Madhav, Shanthi Sabarimurugan, Kartik Lakhotiya, Venkatesh Pandey, T Priyadharshini, Siddhratha Baxi, KM Gothandam, Rama Jayaraj

**Affiliations:** a University of Adelaide, North Terrace Campus, Adelaide South Australia, Australia; b School of Biosciences and Technology, Vellore Institute of Technology (VIT), Vellore; c Department of Biochemistry, Bharathiyar University, Coimbatore, Tamil Nadu, India; d GenesisCare, Bunbury, Western Australia; e College of Health and Human Sciences, Charles Darwin University, Ellengowan drive, Darwin, Australia.

**Keywords:** cancer, diagnosis, HE4, meta-analysis, prognosis, protocol, systematic review

## Abstract

**Background:**

Human epididymis protein 4 (HE4) protein has garnered a great degree of interest as a complementary biomarker to carbohydrate antigen 125 (CA125), or even as an independent biomarker for monitoring, diagnosis, and prognostication of ovarian cancer. Its use is currently limited to ovarian cancer. Recent studies have suggested that it could also be used in other types of cancers.

**Methods:**

The Preferred Reporting Items for Systematic Review and Meta-Analysis Protocols (PRISMA-P) guidelines was used to design this meta-analysis protocol. The final study will also be conducted under the PRISMA guidelines for systematic reviews and meta-analyses. The core bibliographic database search will be carried out by 2 reviewers working individually, with each conducting an initial screening based on titles and abstracts. The shortlisted articles will be selected for review and statistical analysis based on predefined inclusion and exclusion criteria. Study characteristics, relevant clinicopathological characteristics and statistical data required for meta-analysis (hazard ratios [HRs] and 95% confidence interval [CIs) will be extracted and compiled into a MS Excel datasheet. Meta-analysis will be performed, using a random-effects model, and the results (pooled HR and 95% CI) will be presented in the form of a forest plot. Publication bias will also be assessed by use of Egger bias indicator test and funnel plot symmetry. If data are insufficient, a narrative line of review will be pursued.

**Discussion:**

HE4 protein has been shown to have great potential for clinical use as a diagnostic and prognostic marker in epithelial ovarian cancer (EOC). However, HE4 is not only limited to expression in ovarian cancer, but is also overexpressed in lung and endometrial cancers. The effectiveness of HE4 as a biomarker in cancers (other than EOC) has not yet been studied in the form of a comprehensive systematic review and meta-analysis. The results of this study should allow for expanded use of HE4 as a multiutility biomarker in multiple cancer types, thereby, elevating HE4's value as a cancer biomarker.

**PROSPERO registration:**

CRD42019120326.

## 1 Introduction

Human epididymis protein 4 (HE4) is a cancer biomarker that has recently garnered a great degree of interest from the research community. HE4 is a secreted, glycosylated protein that is overexpressed by serous and endometrioid EOCs and is part of the WFDC (previously WAP) family of proteins.^[1]^ HE4 has been suggested as a complement or even as a better alternative to carbohydrate antigen 125 (CA125)^[2–4]^, which was previously being used as a standard diagnostic biomarker and post treatment monitoring tool in ovarian cancer. Although CA125 is a standard biomarker in ovarian cancer, its levels were found to be raised in only 50% of stage 1 epithelial ovarian cancers, and only about 80% in all epithelial ovarian cancers.^[5]^ Therefore, CA125 is not used as an independent marker, but rather in combination with patient data, imaging and a tumor marker profile to differentiate between benign and malignant ovarian cancers.^[6]^ The imaging process (in the case of ovarian cancers, ultrasound) is an integral part of differentiating between benign and malignant tumors, and depends on the expertise of those conducting the imaging.^[7]^ Therefore, CA125 is unable to satisfactorily act as standalone marker for EOC.

In comparison, there are studies suggesting that HE4 could be used as an independent biomarker, in both diagnosis and prognosis of EOC and endometrial cancers.^[8,9]^ It has also been approved by the United States of America (USA), Food and Drug Administration (FDA) as a biomarker to monitor patients with epithelial ovarian cancer.^[10]^ It is observed that in normal tissue, HE4 is restricted to expression by tissue in the reproductive tract and respiratory epithelium.^[11]^ However, HE4 is far from being exclusive to the aforementioned tissue regions. It is also strongly expressed in normal human trachea, salivary glands, lung, prostate, pituitary gland, thyroid, and kidney.^[12]^ Studies have even assessed HE4 as an independent prognostic marker in non-small cell lung cancer (NSCLC).^[13]^

Furthermore, studies assessing HE4 and CA125 as a combined indicator of prognosis have shown positive results, with a sensitivity of 76.4% and specificity of 95%.^[14]^ Although HE4 is already well established as a biomarker for monitoring EOC patients, its potential use in other types of cancer such as endometrial and lung cancer, as well its use as a prognostic and diagnostic marker is still being investigated.

Although there are a number of systematic reviews and meta-analysis studies highlighting the value of HE4 as a prognostic and diagnostic marker in EOC, a comprehensive study that takes into consideration, HE4's multiple use cases and its potential as a biomarker in other relevant cancer types (EOC, lung, and endometrial) has not been performed. This systematic review and meta-analysis protocol details the process behind conducting such a study.

Although the prognostic and diagnostic effect of HE4 in ovarian cancers has been well documented and studied. The effect prognostic effects of HE4 in other cancers, such as endometrial cancer and lung cancer has not been sufficiently investigated.^[15,16]^ The proposed study is designed to ameliorate this issue by conducting a comprehensive systematic review and meta-analysis of all currently published literature on the prognostic and diagnostic utility of HE4 biomarker in relevant cancers. This study is aimed at informing clinical decision making regarding cancer patient prognosis patients (ovarian, endometrial and lung cancer patients in particular), by the use of the HE4 biomarker. This systematic review and meta-analysis will be based on survival data, with the effect size metric analyzed for this study being the hazard ratio (HR).

This study will help determine the prognostic efficacy of HE4 as a biomarker in different cancers. It will serve as a review of all currently published literature on HE4 as a prognostic marker, while also providing a succinct analysis of the quality of the studies, and the overall prognostic efficacy of HE4 expression as is represented across all published clinical studies. The study aims to help clinical decision-making by delineating the magnitude of the effect size (HR), which represents the effectiveness of HE4 in prognosticating each cancer type. As this study is a systematic review and meta-analysis, it will also inform future researchers on the potential research avenues regarding this topic.

## 2 Methods

### 2.1 Search strategy

The systematic review and meta-analysis protocol was designed in accordance to the standardized guidelines established in Preferred Reporting Items for Systematic Reviews and Meta-Analysis Protocols (PRISMA-P).^[17,18]^ The search strategy for proposed study involves an exhaustive search of bibliographic databases. PubMed, MEDLINE, EMBASE, Science Direct, Scopus and Web of Science, literature databases will be searched for articles published between January 1998 and December 2018. The search will be conducted using search strings consisting of the following “keywords” such as, “Human Epididymis protein 4;” “HE4;” “cancer;” “ovarian cancer;” “lung cancer;” “endometrial cancer;” “biomarker;” “prognosis;” “diagnosis;” “patient;” “clinical;” “survival;” “overall survival;” “disease free survival;” “disease specific survival;” “Hazard Ratio;” “Carbohydrate Antigen 125;” “CA125.” The articles screened-in for inclusion into the systematic review and meta-analysis study will have their reference lists screened for further studies that may be suitable for inclusion, thereby increasing the robustness of the search. Two reviewers will independently perform the article search, so as to ameliorate selection bias. The initial selection during search will be based on the screening of titles and abstracts of published studies, wherein the reviewers will assess the suitability of each study, to the topic under review.

### 2.2 Inclusion and exclusion criteria

After initial selection of articles after database search and title/abstract based screening, the selected articles will undergo a rigorous secondary screening process based on strict, predefined inclusion and exclusion criteria. The criteria have been specified below.

Inclusion criteria are as follows:

1. Studies that discuss HE4 in the context of survival outcome in cancer patients.
2. Studies that explicitly report HR and 95% CI values as part of the main manuscript or supplementary data.
3. Articles that were published between 1998 and 2018.
4. Studies that used overall survival (OS) as the survival end-point of the study.
5. Studies that were in accordance with preestablished guidelines for systematic reviews and meta-analysis, such as PRISMA, JBI, and MOOSE, and so on.^[19–21]^
6. Studies that provided sufficient data to extract HR and 95% CI values for OS, if the values were not explicitly stated in the study.

Exclusion criteria:

1. Conference abstracts, reviews and letters to the editor will not be included.
2. Studies reporting results from in-vitro, in-silico or animal studies will be excluded.
3. Theses and incomplete studies will be excluded.
4. Studies that do not report data on HE4 expression in cancer patients will be excluded.
5. Studies that have a patient sample size of less than 10 will not be considered.

There will be no limitations on selection based on pathophysiological and clinical characteristics.

The corresponding authors of articles with missing data will be contacted to obtain the missing data. In case the missing data are obtained and the studies meet the quality criteria, the studies will be included into the systematic review and meta-analysis.

### 2.3 Data extraction and management

The data will be extracted individually from the screened studies by 2 reviewers, and recorded into a standardized data extraction form. After data extraction, the whole dataset will be collated into a single comprehensive Microsoft Excel spreadsheet. The relevant figures, tables, and charts will be collated into a separate database. The following data items will be extracted from the published studies.

1. Name of the first author
2. Year of publication
3. Country
4. Number of participants
5. Study population
6. Assay methods
7. Tumor stage
8. Tumor anatomic location
9. Clinicopathological characteristics (age, sex, risk factors, and metastasis)
10. HE4 expression rate
11. HR with 95% CI of patient survival for, OS, disease-free survival (DFS), and/or disease-specific survival (DSS).

### 2.4 Quality assessment

Two reviewers will individually assess the quality of included studies using a standardized quality appraisal tool. This quality assessment tool has been developed by the National Heart, Lung and Blood Institute (NHLBI) for observational and cross-sectional studies.^[22]^ This assessment tool will be applied to all the selected full-text articles which will be rated as good, fair or poor. If any disagreements between reviewers arise during quality appraisal, a third reviewer will be included to clarify the issue.

### 2.5 Meta-analysis

The meta-analysis will be performed with the aid of the Comprehensive Meta-Analysis software. Forest plots will be generated, pooling the HR and 95% CI values from all selected studies. The analysis of OS, DFS, and DSS will be performed separately, as each indicates a different survival endpoint. Random-effects model will be used to perform the meta-analysis owing to the inherent heterogeneity (owing to differences in study parameters) between individual studies. The heterogeneity between studies will also be evaluated using the Cochran Q Test, the Higgins’ *I*^2^ statistic and the Tau^2^ value.^[23–25]^ The *I*^2^ statistic will be given precedence over the Cochran *Q* because of its higher power of detection of heterogeneity.^[26]^ The Tau^2^ value, however, will indicate the variance of the effect size parameters across the population of studies and will reflect the variance of the true effect sizes.^[25,27,28]^ The *P* value will indicate the overall statistical significance of HE4 as a prognostic marker across all included studies.^[29,30]^ As HR is the effect size metric proposed for this study, the pooled HR and 95% CI value will indicate the magnitude of prognostic efficacy of HE4 in cancer patients.^[31–34]^ Subgroup analysis is predicated on the quantity and quality of statistical data extracted from the selected studies.^[35]^ The tentative subgroups for subgroup analysis are ovarian cancer, endometrial cancer, and lung cancer. If sufficient data are available, subgroup analysis based on sex, location, ethnicity, and risk factors will also be conducted.

### 2.6 Publication bias

Publication bias is a type of bias that is inherent to systematic review and meta-analysis studies.^[36]^ As the journal publication process tends to favor positive results and large studies, oftentimes small studies and negative results are not reported.^[27,37–40]^ Therefore, any study that aims to collate prepublished studies and provide an overall analysis, requires to assess the extent of publication bias that exists in the set of collected studies, so as to avoid bias provide an accurate analysis.^[22,41,42]^ The methods that will be used to assess publication bias are as follows.

A funnel plot will be constructed using standard error (*y*-axis) and log (HR) (*x*-axis), and the studies included for meta-analysis will be plotted onto the graph. Egger bias indicator test will be used to plot a regression line, which will be used to assess the symmetry of the plotted studies and examine for the presence of any publication bias.^[43]^

Orwin Fail-Safe N test will be used to determine missing studies, which may skew the regression line.^[44]^ The missing studies (adjusting for studies that may not have been reported) will be imputed and assessed, with the regression line being shifted to better accommodate imputed studies, and provide a more accurate estimate of publication bias (Table 1).

**Table 1:**
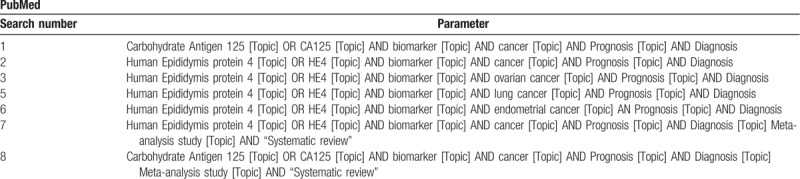
The initial search strategy.

Begg and Mazumdar Rank correlation test will also be used to assess publication bias using the correlation between the ranks of effect sizes and the ranks of their variances.^[45]^ Positive values of this test are indicative of a higher test accuracy.

### 2.7 Reporting of the review

**Figure 1:**
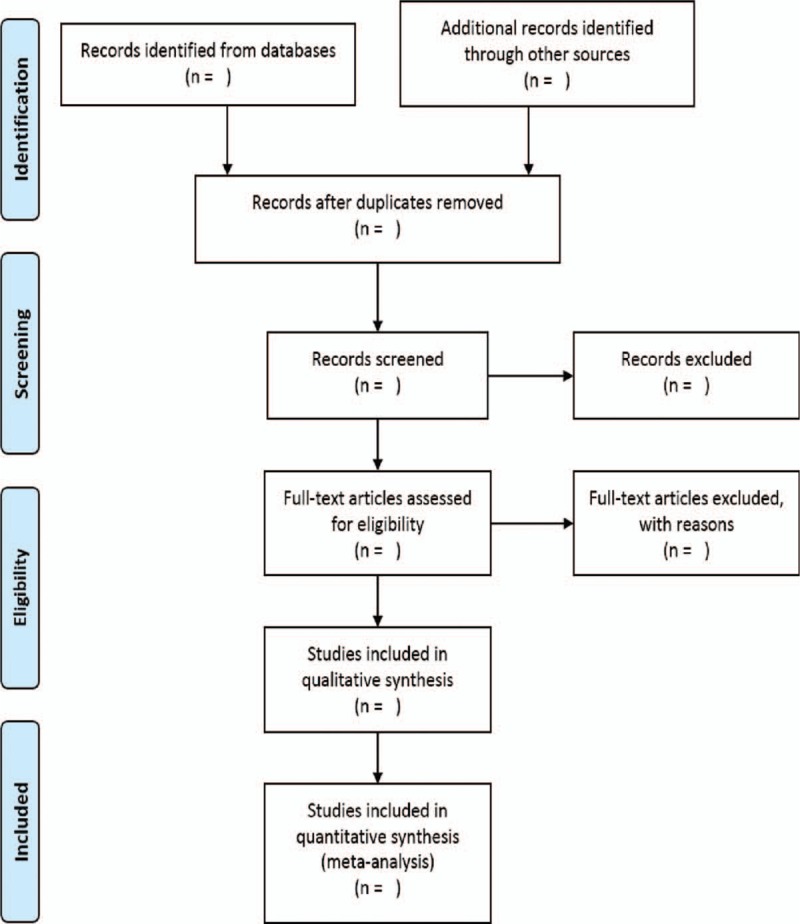
Flow chart for the study selection.

The findings will be published as per PRISMA guidelines.^[19]^ A flow chart will be employed to outline the selection process (Fig. 1). Text description will be used to review the qualitative data of the included studies. Outputs of meta-analyses will be depicted in a forest plot. Publication bias will be represented in the inverted funnel plot. The search strategy and quality appraisal tool will be provided in the supplement.

### 2.8 Ethics and dissemination

This protocol is prepared according to PRISMA-P guidelines. This study will be conducted using publicly available data without involving human participants. Therefore, it does not require formal human research ethics committee review. We plan to publish our findings in peer-reviewed journals and relevant conference proceedings. In addition, we believe the results of the systematic review will have implications for policy and practice. We will prepare policy-maker summary using a validated format, disseminate through social media, and email discussion groups.

## 3 Discussion

A number of research endeavors have been focused toward HE4 as a biomarker for monitoring in ovarian cancer. Present research studies suggest HE4 at minimum as a co-marker to be used alongside CA125, or at best, an independent biomarker capable of individually monitoring EOC patients. A number of meta-analysis studies support the use of HE4 as a marker in ovarian cancer. However, the possibility of use of HE4 in other types of cancer, or as a prognostic marker, has not been adequately explored. Particularly as a systematic review and meta-analysis study, this proposed study may be able to highlight other randomized controlled trial studies which have focused on the use of HE4 in cases beyond that of ovarian cancer. The results of this study should be able to inform if such use of the HE4 marker is a viable clinical option; If HE4 does/does not have sufficient power of detection in cancers other than HE4; if further studies are required before a concrete statement on its utility may be made. The proposed study fills a niche in the wider topic of HE4 as a cancer biomarker, and should help fill the knowledge gap currently present in this topic. There are a few possible limitations that the proposed study may face. The lack of sufficient, high-quality studies to conduct a full meta-analysis may be an issue, in which case a comprehensive literature review (systematic review) will be presented instead. A high level of between-study heterogeneity may also skew the results, which will be accounted for in analysis of heterogeneity and by using the random-effects model for meta-analysis.

The field of cancer biomarkers is rapidly growing, and HE4's effect on ovarian cancer treatment, currently represents a great example of how new powerful biomarkers may change the landscape of cancer monitoring. The full utility of every such biomarker needs to be explored and evaluated. This field is a work in progress, and as the pool of published research in this field continues to expand, this simple and succinct protocol will continue to act as a guideline, through the years, for researchers evaluating the utility of HE4 or other similar cancer biomarkers.

## Acknowledgments

The authors acknowledge the meta-analysis concepts and applications workshop manual by Michael Borenstein for his guidelines on reporting Meta-analysis, subgroup analysis and publication bias, (www.meta-analysis-workshops.com).

## Author contributions

RJ, CK, MRM and SS contributed to the conceptualization, study design, search strategy, protocol development, and review by revising different versions. RJ, KMG, SB were involved in the supervision, ensured the absence of errors and arbitrated in case of disagreement. CK, SS, MRM, KL, VP, and PT engaged in the manuscript writing and analysis. All authors have read and approved the final version of the manuscript.

**Conceptualization:** Rama Jayaraj, Shanthi Sabarimurugan, T Priyadharshini.

**Formal analysis:** Rama Jayaraj.

**Investigation:** Rama Jayaraj, Chellan Kumarasamy, Madurantakam Royam Madhav, Siddhratha Baxi.

**Methodology:** Rama Jayaraj, Chellan Kumarasamy, Madurantakam Royam Madhav, Venkatesh Pandey.

**Project administration:** Rama Jayaraj, Chellan Kumarasamy.

**Resources:** Rama Jayaraj, Chellan Kumarasamy, Madurantakam Royam Madhav, Shanthi Sabarimurugan.

**Software:** Rama Jayaraj, Kartik Lakhotiya.

**Supervision:** Rama Jayaraj, Kartik Lakhotiya, T Priyadharshini, Siddhratha Baxi, K M Gothandam.

**Validation:** Rama Jayaraj, Shanthi Sabarimurugan, T Priyadharshini, K M Gothandam.

**Visualization:** Rama Jayaraj, K M Gothandam.

**Writing – original draft:** Rama Jayaraj, Chellan Kumarasamy, Madurantakam Royam Madhav, Shanthi Sabarimurugan, Kartik Lakhotiya, Venkatesh Pandey, T Priyadharshini, Siddhratha Baxi, K M Gothandam.

**Writing – review & editing:** Rama Jayaraj, Chellan Kumarasamy, Madurantakam Royam Madhav, Shanthi Sabarimurugan, Kartik Lakhotiya, Venkatesh Pandey, T Priyadharshini, Siddhratha Baxi, K M Gothandam.

Rama Jayaraj orcid: 0000-0002-2179-0510.

## References

[1] Bingle L Singleton V Bingle CD The putative ovarian tumour marker gene HE4 (WFDC2), is expressed in normal tissues and undergoes complex alternative splicing to yield multiple protein isoforms. Oncogene 2002;21:2768–73. 11965550 10.1038/sj.onc.1205363

[2] Kalapotharakos G Asciutto C Henic E High preoperative blood levels of HE4 predicts poor prognosis in patients with ovarian cancer. J Ovarian Res 2012;5:20. 22909379 10.1186/1757-2215-5-20 PMC3480899

[3] Hamed EO Ahmed H Sedeek OB Significance of HE4 estimation in comparison with CA125 in diagnosis of ovarian cancer and assessment of treatment response. Diagnost Pathol 2013;8:11. 10.1186/1746-1596-8-11 PMC3621278 23343214

[4] Molina R Escudero JM Augé JM HE4 a novel tumour marker for ovarian cancer: comparison with CA 125 and ROMA algorithm in patients with gynaecological diseases. Tumour Biol 2011;32:1087–95. 21863264 10.1007/s13277-011-0204-3 PMC3195682

[5] Zurawski VR Jr Knapp RC Einhorn N An initial analysis of preoperative serum CA 125 levels in patients with early stage ovarian carcinoma. Gynecol Oncol 1988;30:7–14. 2452773 10.1016/0090-8258(88)90039-x

[6] Van Gorp T Cadron I Despierre E HE4 and CA125 as a diagnostic test in ovarian cancer: prospective validation of the risk of ovarian malignancy algorithm. Br J Cancer 2011;104:863–70. 21304524 10.1038/sj.bjc.6606092 PMC3048204

[7] Van Holsbeke C Daemen A Yazbek J Ultrasound methods to distinguish between malignant and benign adnexal masses in the hands of examiners with different levels of experience. Ultrasound Obstet Gynecol 2009;34:454–61. 19736644 10.1002/uog.6443

[8] Mutz-Dehbalaie I Egle D Fessler S HE4 is an independent prognostic marker in endometrial cancer patients. Gynecol Oncol 2012;126:186–91. 22525819 10.1016/j.ygyno.2012.04.022

[9] Hellström I Raycraft J Hayden-Ledbetter M The HE4 (WFDC2) protein is a biomarker for ovarian carcinoma. Cancer Res 2003;63:3695–700. 12839961

[10] Montagnana M Danese E Giudici S HE4 in ovarian cancer: from discovery to clinical application. Adv Clin Chem 2011;55:2. 22126021

[11] Drapkin R von Horsten HH Lin Y Human epididymis protein 4 (HE4) is a secreted glycoprotein that is overexpressed by serous and endometrioid ovarian carcinomas. Cancer Res 2005;65:2162–9. 15781627 10.1158/0008-5472.CAN-04-3924

[12] Galgano MT Hampton GM Frierson HF Jr Comprehensive analysis of HE4 expression in normal and malignant human tissues. Mod Pathol 2006;19:847–53. 16607372 10.1038/modpathol.3800612

[13] Lamy P-J Plassot C Pujol J-L Serum HE4: an independent prognostic factor in non-small cell lung cancer. PLoS One 2015;10:e0128836. 26030627 10.1371/journal.pone.0128836 PMC4452338

[14] Moore RG Brown AK Miller MC The use of multiple novel tumor biomarkers for the detection of ovarian carcinoma in patients with a pelvic mass. Gynecol Oncol 2008;108:402–8. 18061248 10.1016/j.ygyno.2007.10.017

[15] Yu S Yang HJ Xie SQ Diagnostic value of HE4 for ovarian cancer: a meta-analysis. Clin Chem Lab Med 2012;50:1439–46. 22868811 10.1515/cclm-2011-0477

[16] Huang J Chen J Huang Q Diagnostic value of HE4 in ovarian cancer: A meta-analysis. Eur J Obstet Gynecol Reprod Biol 2018;231:35–42. 30317143 10.1016/j.ejogrb.2018.10.008

[17] Moher D Shamseer L Clarke M Preferred reporting items for systematic review and meta-analysis protocols (PRISMA-P) 2015 statement. Syst Rev 2015;4:1. 25554246 10.1186/2046-4053-4-1 PMC4320440

[18] Kumarasamy C Devi A Jayaraj R Prognostic value of microRNAs in head and neck cancers: a systematic review and meta-analysis protocol. Syst Rev 2018;7:150. 30285880 10.1186/s13643-018-0812-8 PMC6169036

[19] Moher D Liberati A Tetzlaff J Preferred reporting items for systematic reviews and meta-analyses: the PRISMA statement. Ann Intern Med 2009;151:264–9. 19622511 10.7326/0003-4819-151-4-200908180-00135

[20] Peters M Godfrey C McInerney P The Joanna Briggs Institute Reviewers’ Manual 2015: Methodology for JBI Scoping Reviews. Adelaide, SA, Australia: The Joanna Briggs Institute; 2015.

[21] Stroup DF Berlin JA Morton SC Meta-analysis of observational studies in epidemiology: a proposal for reporting. Meta-analysis Of Observational Studies in Epidemiology (MOOSE) group. Jama 2000;283:2008–12. 10789670 10.1001/jama.283.15.2008

[22] Sabarimurugan S Madurantakam Royam M Das A Systematic review and meta-analysis of the prognostic significance of miRNAs in melanoma patients. Mol Diagn Ther 2018;22:653–69. 30259393 10.1007/s40291-018-0357-5

[23] Cochran WG The comparison of percentages in matched samples. Biometrika 1950;37:256–66. 14801052

[24] Higgins JPT Thompson SG Deeks JJ Measuring inconsistency in meta-analyses. BMJ 2003;327:557–60. 12958120 10.1136/bmj.327.7414.557 PMC192859

[25] Deeks JJ Higgins JPT Altman DG Analysing data and undertaking meta-analyses. Cochrane handbook for systematic reviews of interventions: Cochrane book series 2008 243–96.

[26] Huedo-Medina TB Sanchez-Meca J Marin-Martinez F Assessing heterogeneity in meta-analysis: Q statistic or I2 index? Psychol Methods 2006;11:193–206. 16784338 10.1037/1082-989X.11.2.193

[27] Jayaraj R Kumarasamy C Sabarimurugan S Commentary: blood-derived microRNAs for pancreatic cancer diagnosis: a narrative review and meta-analysis 2018;9:1896. 10.3389/fphys.2018.01896 PMC6350410 30723420

[28] Jayaraj R Kumarasamy C Systematic review and meta-analysis of cancer studies evaluating diagnostic test accuracy and prognostic values: approaches to improve clinical interpretation of results. Cancer Manag Res 2018;10:4669–70. 30410400 10.2147/CMAR.S183181 PMC6199231

[29] Jayaraj R Kumarasamy C Letter to the editor about the article:“ Performance of different imaging techniques in the diagnosis of head and neck cancer mandibular invasion: a systematic review and meta-analysis”. J Oral Oncol 2019;89:159–60. 10.1016/j.oraloncology.2018.12.024 30606668

[30] Jayaraj R Kumarasamy C Ramalingam S Systematic review and meta-analysis of risk-reductive dental strategies for medication related osteonecrosis of the jaw among cancer patients: approaches and strategies. Oral Oncol 2018;86:312–3. 30262125 10.1016/j.oraloncology.2018.09.017

[31] Coe R Annual Conference of the British Educational Research Association, University of Exeter, England, 12–14 September 2002.

[32] Jayaraj R Kumarasamy C Gothandam KM Letter to the editor “Prognostic value of microRNAs in colorectal cancer: a meta-analysis”. Cancer Manag Res 2018;10:3501–3. 30271198 10.2147/CMAR.S177875 PMC6145637

[33] Poddar A Aranha RR G KM Head and neck cancer risk factors in India: protocol for systematic review and meta-analysis. BMJ Open 2018;8:e020014. 10.1136/bmjopen-2017-020014 PMC6104749 30127047

[34] Jayaraj R Kumarasamy C Piedrafita D Systematic review and meta-analysis protocol for Fasciola DNA vaccines. Online J Vet Res 2018;22:517.

[35] Jayaraj R Kumarasamy C Madurantakam Royam M Letter to the editor: is HIF-1alpha a viable prognostic indicator in OSCC? A critical review of a meta-analysis study. World J Surg Oncol 2018;16:111. 29914529 10.1186/s12957-018-1408-4 PMC6006741

[36] Jayaraj R Kumarasamy C Sabarimurugan S Letter to the Editor in response to the article,“ The epidemiology of oral human papillomavirus infection in healthy populations: a systematic review and meta-analysis”. Oral Oncol 2018;84:121–2. 30075910 10.1016/j.oraloncology.2018.07.018

[37] Jayaraj R Kumarasamy C Comment on,“ Survival for HPV-positive oropharyngeal squamous cell carcinoma with surgical versus non-surgical treatment approach: a systematic review and meta-analysis”. J Oral Oncol 2018;90:137–8. 10.1016/j.oraloncology.2018.12.019 30579681

[38] Jayaraj R Kumarasamy C Conceptual interpretation of analysing and reporting of results on systematic review and meta-analysis of optimal extent of lateral neck dissection for well-differentiated thyroid carcinoma with metastatic lateral neck lymph nodes. Oral Oncol 2019;89:153–4. 30612934 10.1016/j.oraloncology.2018.12.031

[39] Jayaraj R Kumarasamy C Samiappan S Letter to the Editor regarding, “The prognostic role of PD-L1 expression for survival in head and neck squamous cell carcinoma: a systematic review and meta-analysis”. Oral Oncol 2019;90:90–139. 10.1016/j.oraloncology.2018.12.018 30579680

[40] Jayaraj R Kumarasamy C Comment on ’Prognostic biomarkers for oral tongue squamous cell carcinoma: a systematic review and meta-analysis’. Br J Cancer 2018;118:e11. 10.1038/bjc.2017.482 PMC5846075 29449675

[41] Jayaraj R Kumarasamy C Madhav MR Comment on “systematic review and meta-analysis of diagnostic accuracy of miRNAs in patients with pancreatic cancer”. Dis Markers 2018;2018:6904569. 30425753 10.1155/2018/6904569 PMC6217755

[42] Madhav MR Nayagam SG Biyani K Epidemiologic analysis of breast cancer incidence, prevalence, and mortality in India: protocol for a systematic review and meta-analyses. Medicine (Baltimore) 2018;97:e13680. 30593138 10.1097/MD.0000000000013680 PMC6314759

[43] Egger M Smith GD Schneider M Bias in meta-analysis detected by a simple, graphical test. Bmj 1997;315:629–34. 9310563 10.1136/bmj.315.7109.629 PMC2127453

[44] Orwin RG A fail-safe N for effect size in meta-analysis. J Educ Stat 1983;8:157–9.

[45] Begg CB Mazumdar M Operating characteristics of a rank correlation test for publication bias. Biometrics 1994;50:1088–101. 7786990

